# Biomechanical Evaluation of Tarsometatarsal Fusion Comparing Crossing Lag Screws and Lag Screw With Locking Plate

**DOI:** 10.1177/10711007211033541

**Published:** 2021-08-05

**Authors:** Sarah Ettinger, Lisa-Christin Hemmersbach, Michael Schwarze, Christina Stukenborg-Colsman, Daiwei Yao, Christian Plaass, Leif Claassen

**Affiliations:** 1 Department of Orthopedic Surgery, Hannover Medical School at Diakovere Annastift, Hannover, Germany; 2 Hannover Medical School, Hannover, Germany; 3 Laboratory for Biomechanics and Biomaterials, Hannover Medical School, Hannover, Germany

**Keywords:** tarsometatarsal arthritis, Lisfranc, tarsometatarsal arthrodesis fusion, locking plate

## Abstract

**Background:**

Tarsometatarsal (TMT) arthrodesis is a common operative procedure for end-stage arthritis of the TMT joints. To date, there is no consensus on the best fixation technique for TMT arthrodesis and which joints should be included.

**Methods:**

Thirty fresh-frozen feet were divided into one group (15 feet) in which TMT joints I-III were fused with a lag screw and locking plate and a second group (15 feet) in which TMT joints I-III were fused with 2 crossing lag screws. The arthrodesis was performed stepwise with evaluation of mobility between the metatarsal and cuneiform bones after every application or removal of a lag screw or locking plate.

**Results:**

Isolated lag-screw arthrodesis of the TMT I-III joints led to significantly increased stability in every joint (*P* < .05). Additional application of a locking plate caused further stability in every TMT joint (*P* < .05). An additional crossed lag screw did not significantly increase rigidity of the TMT II and III joints (*P* > .05). An IM screw did not influence the stability of the fused TMT joints. For TMT III arthrodesis, lag-screw and locking plate constructs were superior to crossed lag-screw fixation (*P* < .05). TMT I fusion does not support stability after TMT II and III arthrodesis.

**Conclusion:**

Each fixation technique provided sufficient stabilization of the TMT joints. Use of a lag screw plus locking plate might be superior to crossed screw fixation. An additional TMT I and/or III arthrodesis did not increase stability of an isolated TMT II arthrodesis.

**Clinical Relevance:**

We report the first biomechanical evaluation of TMT I-III arthrodesis. Our results may help surgeons to choose among osteosynthesis techniques and which joints to include in performing arthrodesis of TMT I-III joints.

## Introduction

The tarsometatarsal (TMT) joint line can be divided into 3 columns, including a medial (first TMT joint), central (second and third TMT joint), and lateral (fourth and fifth TMT joint) column. For primary or posttraumatic TMT arthritis, TMT arthrodesis of the medial and central column (I-III) is a standard operative procedure.^11,17^ Despite significant postoperative improvement in foot function and pain, nonunion remains a main complication after TMT fusion, with nonunion rates from 7% to 10%.^3,6,13^ Several clinical and biomechanical studies have focused on different fixation techniques for the first TMT arthrodesis, mostly demonstrating superiority of locking plates and lag screws compared with isolated crossed screw or plate fixation.^2,7,8^ However, there is only limited clinical and biomechanical data for TMT arthrodesis of the medial and central column. Only 2 recent clinical studies focused on the fixation technique for TMT I-III arthrodesis.^3,5^ Buda et al^3^ concluded that isolated plate fixation significantly increased the nonunion rate after midfoot arthrodesis compared to crossed screw fixation. Ettinger et al^5^ found a lower nonunion rate using locking plate plus screw fixation. Two previous biomechanical studies examined midfoot fusion for neuroarthropathic feet and for ligamentous Lisfranc joint injuries.^1,8,12^ However, neither of these studies focused on recent fixation techniques for arthritis of the TMT joints. There is no biomechanical study that compares current common fixation techniques using crossed screws or locking plates with lag screws. Further, it remains unclear to what extent the number of fused TMT joints stabilize the adjacent, nonfused TMT joints.

The present study compares crossed lag screws with locking plate and lag screw for TMT I-III fusion. The aim of this study was to determine a superior fixation construct with respect to stability for TMT I-III arthrodesis. In addition, stability in relation to the number of fused TMT joints was analyzed.

## Material and Methods

Thirty fresh-frozen cadaver feet (Science Care, Phoenix, AZ, USA) were used for this biomechanical study. None of the feet showed any relevant deformities or signs of previous surgery. The mean age of the donors was 75.1 ± 11.4 years. Twelve left and 18 right feet were prepared. Twelve donors were female. All feet were randomly assigned to 2 groups (groups 1 and 2) with 15 feet each. There were no demographic differences between the 2 groups.

All feet were thawed at room temperature before experiments. The tibiae were embedded in a 2-component polyurethane casting resin at the proximal ends (Rencast FC52/53, Huntsman Corp, The Woodlands, TX). The Achilles, posterior tibial, and peroneal tendons were prepared proximal to the ankle joint and fixed with 1 clamp each. The biomechanical setup and testing were performed according to a previously published protocol, which evaluated naviculocuneiform fixation techniques.^9^ The specimens were placed in a custom-made frame with the heel on the ground in neutral position, enabling the application of 300 N axial loading and 10 N traction to each respective tendon in neutral position of the ankle joint.

**Figure 1.**
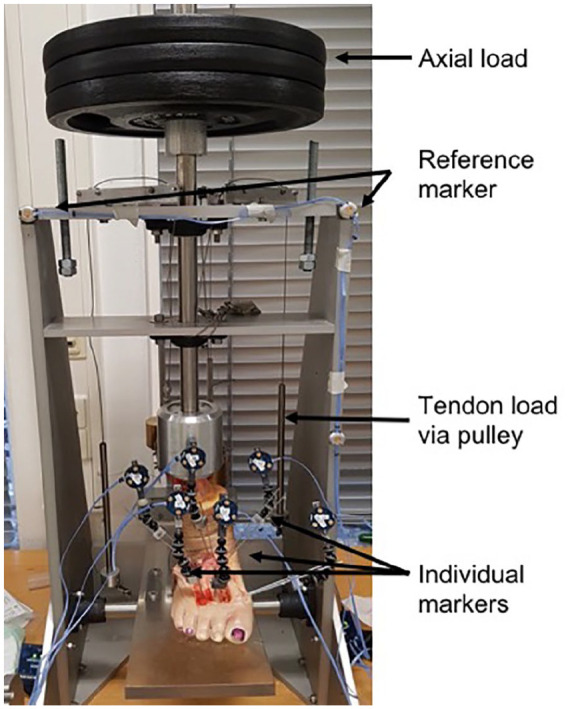
Biomechanical testing setup for tarsometatarsal fusion. The cadaver feet were applied to a frame, enabling the application axial loading and traction to each tendon (tibialis posterior and Achilles tendon, peroneal tendons) in the neutral position of the ankle joint. Individual markers were inserted in the metatarsal bones I-III and in the cuneiform bones I-III. A reference marker was attached to the frame.

For rotational evaluation of the metatarsal (MT) and cuneiform (CN) bones against each other, the NDI Optotrak Certus System (NDI, Ontario, Canada) was used. This system performs an optical measurement with active markers with a resolution of 0.01 mm. Three reference markers were attached to the frame forming a coordinate system fixed in space. Individual marker clusters consisting of 3 markers each were inserted in the MTI-III and the CN I-III via Kirschner wires. The total angle of rotation was measured from the 3 rotational components as the length of the rotation vector between 2 bones (Figure 1).^9^

Biomechanical testing of each group was performed in the same manner. Every operative step was first evaluated without any tendon pull or axial loading (mode 1). Afterward, the same operative step was evaluated with an application of 300-N axial loading on the shank and 10-N tendon pull force each for the posterior tibial tendon, the peroneal tendons, and the Achilles tendon (mode 2). The amount of rotation between mode 1 and 2 for each joint (TMT I-III and the intercuneiform I/II and II/III joints) was the primary outcome parameter. A decreasing amount of rotation was defined as an increased stability of the arthrodesis. To increase reliability of the results, modes 1 and 2 were performed in every group at each operative step to prevent perturbation of the inserted markers during implant application or removal. All procedures were performed by an experienced foot and ankle surgeon.

**Figure 2.**
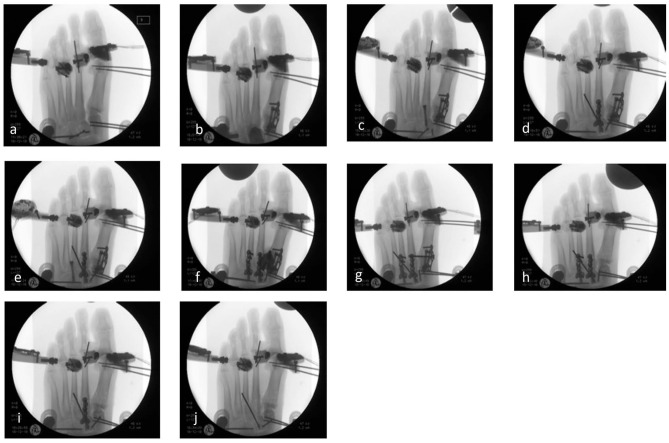
Tarsometatarsal (TMT) fusion using locking plate plus lag screw. (A) The first measurement was performed in the natural state without any arthrodesis. (B) The first TMT joint was fused, using a locking plate plus lag screw. (C-F) Next, the second and third TMT joints were fused one by one, using a lag screw as a first step and, second, an additional locking plate. (G) Subsequently, an intermetatarsal (IM) screw was inserted. The implants were removed step by step, beginning with the TMT I joint and the IM screw (H), then the TMT III joint (I), and finally the TMT II joint (J).

**Figure 3.**
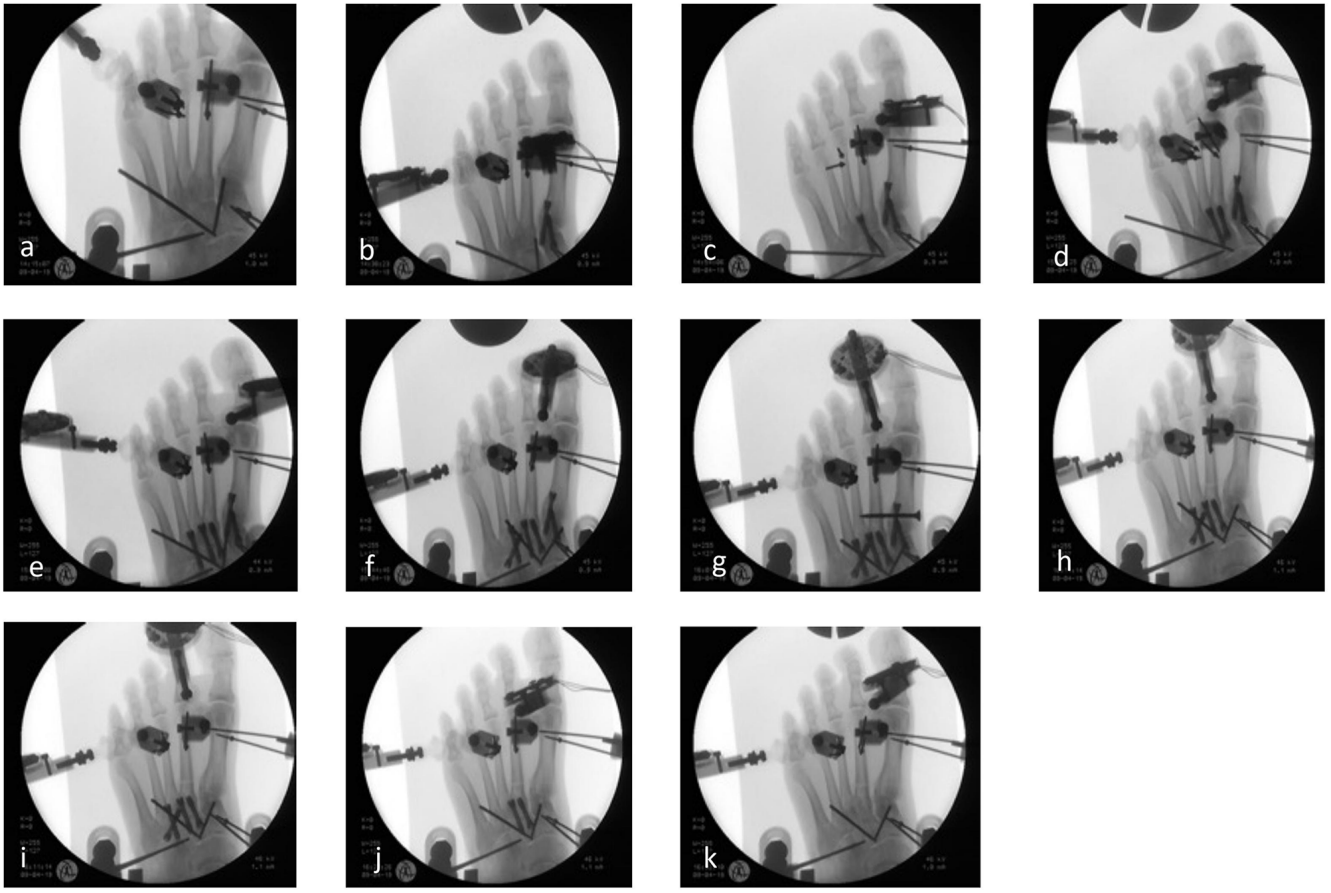
Tarsometatarsal (TMT) fusion using 2 crossed lag screws. (A) The first measurement was performed in the natural state without any arthrodesis. (B) The first TMT joint was fused, using 2 crossed lag screws. (C-F) Next, the second and third TMT joint were fused one by one, using one crossed lag screw as a first step and second, 2 crossed lag screws. (G) Subsequently, an intermetatarsal (IM) screw was inserted. The implants were removed step by step, beginning with the TMT I joint and the IM screw (H), then the TMT III joint (I), and finally the TMT II joint (J).

Arthrodesis was performed using Aptus 2.8 TriLock plates and CCS 5.0 cannulated double-threaded lag screws (Medartis AG, Basel, Switzerland). Prior to stepwise TMT arthrodesis, the first measurement was performed in the native state to define a baseline for every cadaver foot. The stepwise TMT arthrodeses were performed in the following manner: With the markers inserted, the TMT I-III joints were prepared. In group 1, first a CCS 5.0 double-threaded lag screw and second an Aptus 2.8 TriLock plate were inserted separately in each TMT joint I-III, beginning with the first TMT joint (Figure 2). In group 2, first a CCS 5.0 double-threaded lag screw and second a further crossed CCS 5.0 double-threaded lag screw was inserted in each TMT I-III joint, beginning with the first TMT joint (Figure 3). With TMT joints I-III fixed, a I-II intermetatarsal (IM) lag screw was inserted in both groups, directed from the basis of the first MT to the basis of the second MT bone.

Then, all implants were removed step by step, beginning with the IM screw and TMT I arthrodesis to evaluate residual stability of the TMT II and III arthrodeses and second, the TMT III arthrodesis to evaluate residual stability of the isolated TMT II arthrodesis. After removal of the implants in TMT III arthrodesis, each foot was measured again in the natural state without any arthrodesis to provide a comparison to the initial natural conditions. Every step was recorded radiographically to ensure correct implant positioning (Figures 2 and 3). Table 1 presents an overview of every single condition of the respective fused joints.

**Table 1.** Overview of Single Conditions for the Respective Fused Joints.^a^

Condition	TMT I		TMT II		TMT III		MT I /MT II
	Screw	Plate + Screw /Crossed Screws	Screw	Plate + Screw /Crossed Screws	Screw	Plate + Screw /Crossed Screws	IM Screw
01							
02	X	X					
03a	X	X	X				
03b	X	X	X	X			
04a	X	X	X	X	X		
04b	X	X	X	X	X	X	
05	X	X	X	X	X	X	X
06			X	X	X	X	
07			X	X			
08							

a Stepwise arthrodeses of the tarsometatarsal (TMT) joints were performed, using an isolated lag screw (conditions 03a, 04a), lag-screw plus plate constructs, or 2 lag screws (conditions 02, 03b, and 04b). With the TMT joints I-III fixed, an intermetatarsal (IM) I/II screw was added (condition 05). Next, the implants were gradually removed, beginning with the first TMT joint and IM screw (condition 06), followed by the second TMT joint (condition 07), and finally the third TMT joint with all implants removed (condition 08).

### Statistical Analysis

Data collection and analysis was conducted in R (version 4.0.3, R Core Team, Vienna, Austria). Values are expressed as median and range. The statistical analysis was performed using a Wilcoxon signed-rank test for paired data on an interval scale, that is comparison of the different conditions. A Mann–Whitney *U* test was used to compare the 2 fixation techniques. Statistical significance was defined as a *P* value <.05.

## Results

### Lag Screw and Locking Plate

**Figure 4.**
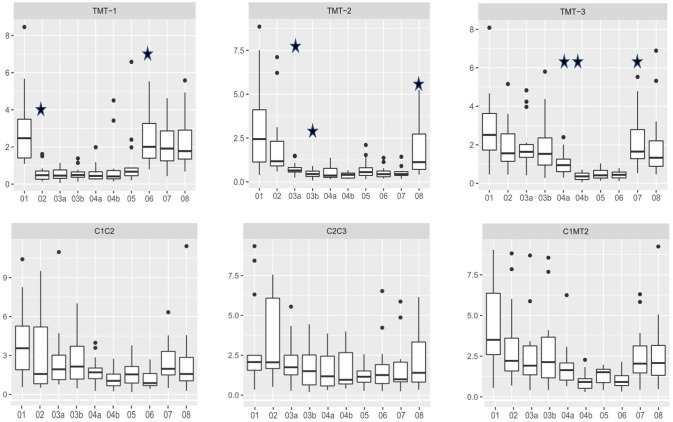
Measurement of rotational movement of the tarsometatarsal and intercuneiform joints during stepwise arthrodesis by 1 lag screw plus locking plate. Every step that led to significantly reduced movement in the respective joint is marked with a star (*P* < .05).

The application of a locking plate and lag screw for TMT I arthrodesis led to significantly increased stability of the first TMT joint (Figure 4; condition 01 vs 02; *P* < .001). A TMT I arthrodesis did not lead to additional rigidity of the second and third TMT joint.

Applying a single lag screw for TMT II arthrodesis significantly increased the stability of the second TMT joint (Figure 4; condition 02 vs 03a; *P* < .001). With an additional locking plate, further significant increase in stability was detected (Figure 4; condition 03a vs 03b; *P* = .007). Removal of TMT I and III arthrodeses did not decrease the rigidity of the TMT II arthrodesis.

The application of a single lag screw for TMT III arthrodesis significantly increased the stability of the third TMT joint (Figure 4; condition 03b vs 04a; *P* = .003). With an additional locking plate, stability was further significantly increased (Figure 4; condition 04a vs 04b; *P* < .001). Removal of the TMT I arthrodesis did not decrease the rigidity of the TMT III arthrodesis.

A I-II IM screw did not lead to additional stability in any of the TMT joints, nor in the intercuneiform or intermetatarsal joints (Figure 4; condition 04b vs 5; *P* > .05).

Removal of each implant led to increased mobility in the respective joints, comparable to the initial values (*P* < .05).

### Crossed Lag Screws

**Figure 5.**
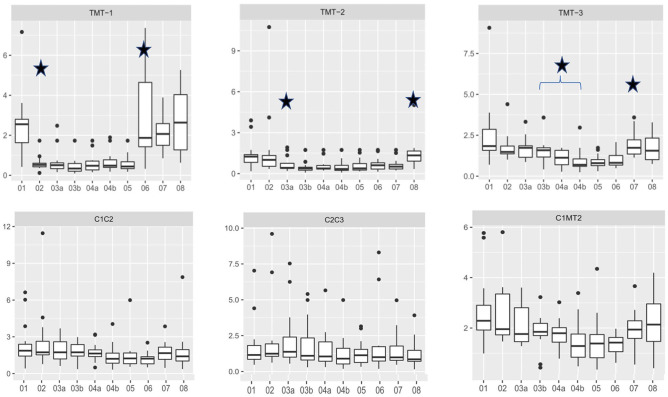
Measurement of rotational movement of the tarsometatarsal and intercuneiform joints during stepwise arthrodesis by 2 crossed lag screws. Every step that led to significantly reduced movement in the respective joint is marked with a star (*P* < .05).

The application of 2 crossed lag screws for TMT I arthrodesis led to significantly increased stability of the first TMT joint (Figure 5; condition 01 vs 02; *P* < .001). A TMT I arthrodesis did not lead to additional rigidity of the second and third TMT joints.

Applying a single lag screw for TMT II arthrodesis led to significantly increased stability of the second TMT joint (Figure 5; condition 02 vs 03a; *P* = .0067). With an additional crossed lag screw, there was an increase in stability that was not statistically significant (Figure 5; condition 03a vs 03b; *P* = .064). Removal of TMT I and III arthrodeses did not decrease the rigidity of the TMT II arthrodesis.

The application of a single lag screw for TMT III arthrodesis led to increased stability of the third TMT joint that was not statistically significant (Figure 5; condition 02 vs 03a; *P* = .064). With an additional crossed screw, there was a small increase in stability but it was not statistically significant (Figure 5; condition 04a vs 04b; *P* = .1688). Compared with the initial values of the TMT III joint, an arthrodesis of 2 crossed screws significantly increased stability (Figure 5; condition 03b vs 04b; *P* < .001). Removal of the TMT I arthrodesis did not decrease the rigidity of the TMT III arthrodesis.

A I-II IM screw did not lead to additional stability in any of the TMT joints, nor the intercuneiform or intermetatarsal joints (Figure 5; condition 04b vs 5; *P* > .05).

Removal of each implant led to increased mobility in the respective joints, comparable to the initial values (*P* < .05).

### Lag Screw and Locking Plate vs Crossed Screws

**Figure 6.**
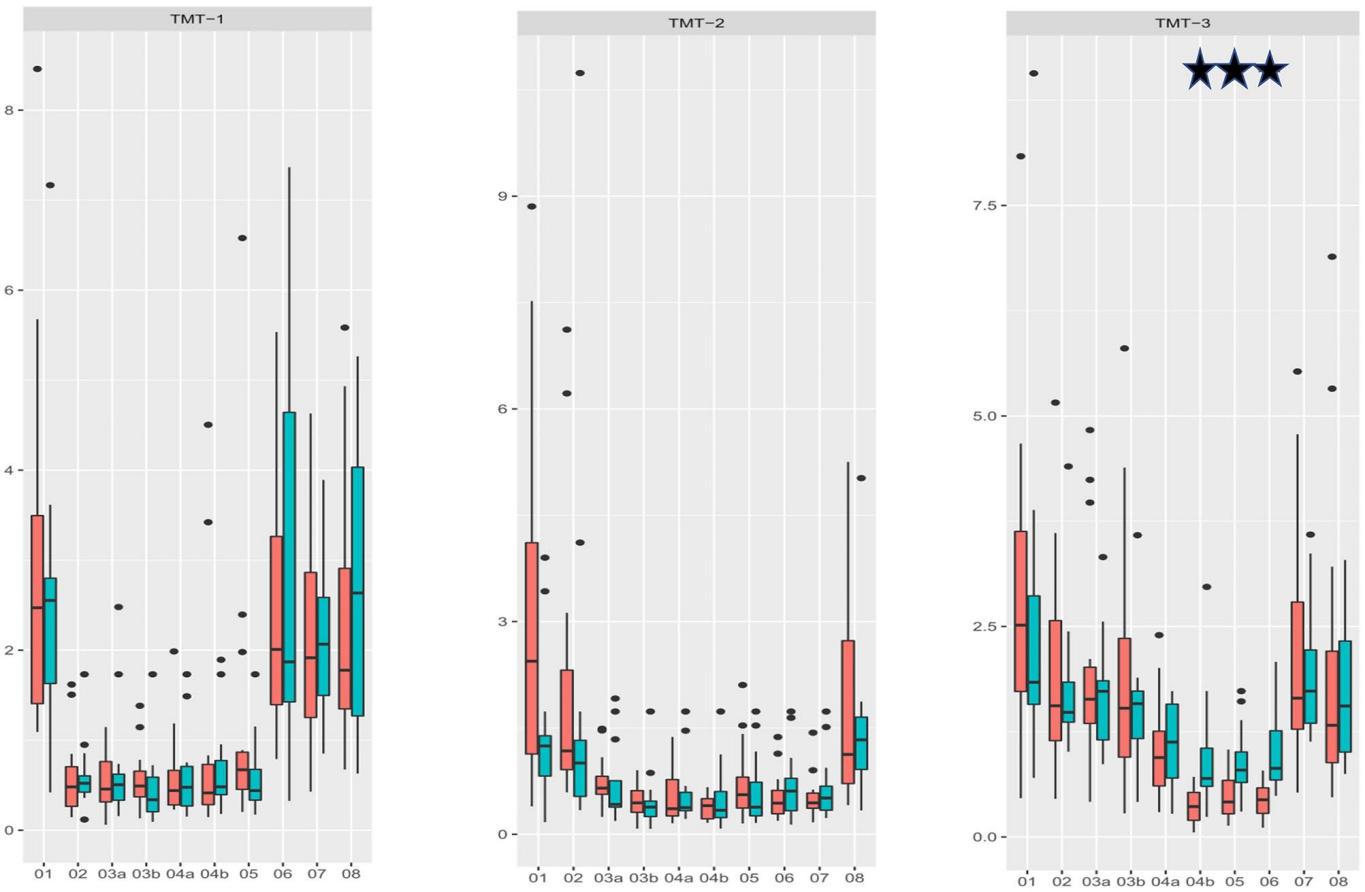
Comparison of lag-screw plus plate constructs with 2 crossed lag screws. For TMT I and II joint arthrodesis, neither of the 2 techniques were superior (*P* = .964; *P* = .304). For TMT III arthrodesis, lag-screw plus locking plate constructs showed significantly higher stability compared to 2 crossed lag screws (*P* < .001). Significant differences are marked with a star.

For the first and second TMT arthrodeses, there was no significant difference in stability between the 2 fixation techniques (Figure 6; *P* = .964, *P =* .304). For TMT III arthrodesis, lag-screw and locking plate constructs showed significantly higher stability compared with crossed lag-screw fixation (Figure 6; *P* = .0007).

## Discussion

Different techniques for midfoot arthrodesis have been investigated. To our knowledge, this biomechanical study is the first comparing crossed lag screws with locking plate and lag-screw constructs for midfoot arthrodesis of the medial and central column. Our data showed that isolated lag-screw fixation was significantly inferior to 2 lag screws or lag-screw and locking plate constructs. For TMT III arthrodesis, lag-screw and locking plate constructs were superior to crossed lag-screw fixation. An additional TMT I and/or III arthrodesis did not increase the stability of an isolated TMT II arthrodesis.

Many previous biomechanical studies focused on the first TMT joint,^3,8,14-16^ the majority stating that locking plate and lag-screw constructs are superior to isolated lag-screw fixation. Klos et al^8^ compared medial with plantar locking plates and showed higher stiffness for plantar plating. Clinical studies have confirmed these biomechanical findings and reported a reduced nonunion rate with locking plates and lag-screw constructs.^4,7^ In our experiments, we used a medial locking plate for TMT I arthrodesis. For TMT I, we could not detect a significant difference compared to crossed lag-screw fixation, which may be due to the smaller axial load of 300 N and the lack of load-to-failure testing.

Two recent clinical studies focused on midfoot arthrodesis. Buda et al^3^ reported a higher nonunion rate with isolated bridge plating without a lag screw (11.4% vs 4.8%), whereas Ettinger et al^5^ reported a lower nonunion rate after locking plate plus compression-screw fixation compared to 2 crossed screws fixation. Two previously performed biomechanical studies focused on fixation of the first and second TMT joints.^1,12^ Marks et al^12^ reported higher loads-to-failure of a plantar plate and lag-screw construct compared to isolated screw constructs. In our study, TMT joints II and III were initially fixed with an isolated lag screw in both groups. Application of an additional locking plate led to significantly increased stability in the second and third TMT joints in group 1, whereas an additional lag screw did not significantly decrease movement in the respective TMT joints in group 2. This might indicate higher rigidity of locking plate and lag-screw constructs. However, we could detect the superiority of locking plate and lag-screw constructs only for the third TMT joint (*P* < .05).

In our experiments, an additional I-II IM screw did not lead to further stability in any of the analyzed joints. One recent study by Langan et al^10^ compared TMT I arthrodesis constructs with or without an additional I-II IM screw and reported a greater radiologically improved IM angle and hallux valgus angle in patients with an additional I-II IM screw. In their cohort, the TMT I arthrodesis was performed with a locking plate and cross screw from MT I into intermediate cuneiform constructs instead of a screw fixation of MT I and II. Our testing setup did not focus on the IM and hallux valgus angles, which might have improved after the I-II IM screw as well. Further, a I-II intercuneiform screw or cross screw might have decreased movement in the respective joints in our testing. Biomechanical and clinical studies should be performed to analyze the effects of an intercuneiform screw.

Some patients have isolated TMT II, TMT III, or combined TMT II and III arthritis. To our knowledge, there is no previous study analyzing TMT stability in relation to the number of fused TMT joints. In our analysis, stability of the TMT II arthrodesis remained constant, independent of whether an additional TMT I or III arthrodesis existed. Further, an additional TMT I arthrodesis did not influence the stability of a TMT III arthrodesis. Thus, isolated TMT II or TMT II and III arthrodeses seem to have no adverse effects on stability. These findings are of high clinical relevance and indicate that isolated TMT II or III fusion may not increase risk of nonunion. So, in cases of an unaffected TMT I joint, the TMT I can be left intact and does not have to be fused for stability. Appropriately, Buda et al^3^ stated in their clinical study that the number of treated columns did not affect the nonunion rate, which supports our findings. However, in their cohort, they did not distinguish between the type of TMT arthrodesis performed (TMT I-III, I-II, II-III, etc). Further comparative clinical studies are needed to support our findings.

Our study has some limitations that have to be addressed. First, this was a cadaver study. Differences in bone quality might have biased our results. Compared with other biomechanical studies, we did not perform maximum-load-to-failure testing. Other than analysis and comparison of 2 different fixation techniques, our study design evaluated effects on stability dependent on the number of fused TMT joints, according to a previously published protocol.^9^ Our procedures were designed to ensure stepwise measurements of the performed arthrodesis. Thus, we did not do load-to-failure testing. A randomization of the fixation sequence to counter possible ordering effects was not deemed feasible, because it could result in a test order in which a single screw would have to be removed and installed multiple times, which would have drastically affected its stability and subsequently introduced additional bias. An additional testing with the heel off could have shown greater impact on the TMT movements, as this means the greatest loads on the TMT joint line. Third, the utilized setup captured only rotational motion and not translational motion. However, because of the loading of the specimen and the type of fixation, predominantly rotational motion can be expected. At last, we performed axial loading with only 300 N, which is not comparable to physiological loads in vivo. In pilot studies, a higher axial load (800 N) led to wearing out of the cadaver feet throughout the testing protocol. Further, no differences were detected in the TMT joint rotation ratios comparing axial loading with 800 and 300 N. With a reduced load and additive tendon pull, the final measurements differed only minimally from the baseline measurement, which confirmed that our specimens were not worn out.

## Conclusion

Isolated lag-screw fixation was significantly inferior to 2 lag screws or lag-screw and locking plate constructs. An additional TMT I and/or III arthrodesis did not increase the stability of an isolated TMT II arthrodesis.

## Supplemental Material

sj-pdf-1-fai-10.1177_10711007211033541 – Supplemental material for Biomechanical Evaluation of Tarsometatarsal Fusion Comparing Crossing Lag Screws and Lag Screw With Locking Plate Click here for additional data file. Supplemental material, sj-pdf-1-fai-10.1177_10711007211033541 for Biomechanical Evaluation of Tarsometatarsal Fusion Comparing Crossing Lag Screws and Lag Screw With Locking Plate by Sarah Ettinger, Lisa-Christin Hemmersbach, Michael Schwarze, Christina Stukenborg-Colsman, Daiwei Yao, Christian Plaass and Leif Claassen in Foot & Ankle International
